# Aerosol Transmission of Filoviruses

**DOI:** 10.3390/v8050148

**Published:** 2016-05-23

**Authors:** Berhanu Mekibib, Kevin K. Ariën

**Affiliations:** 1 Virology Unit, Department of Biomedical Sciences, Institute of Tropical Medicine, Nationalestraat 155, Antwerp B-2000, Belgium; berhanumm2002@gmail.com; 2 School of Veterinary Medicine, College of Natural and Computational Sciences, Hawassa University, P.O. Box 05, Hawassa, Ethiopia

**Keywords:** Ebola, aerosol transmission, pigs, veterinary medicine

## Abstract

Filoviruses have become a worldwide public health concern because of their potential for introductions into non-endemic countries through international travel and the international transport of infected animals or animal products. Since it was first identified in 1976, in the Democratic Republic of Congo (formerly Zaire) and Sudan, the 2013–2015 western African Ebola virus disease (EVD) outbreak is the largest, both by number of cases and geographical extension, and deadliest, recorded so far in medical history. The source of ebolaviruses for human index case(s) in most outbreaks is presumptively associated with handling of bush meat or contact with fruit bats. Transmission among humans occurs easily when a person comes in contact with contaminated body fluids of patients, but our understanding of other transmission routes is still fragmentary. This review deals with the controversial issue of aerosol transmission of filoviruses.

## 1. The Family *Filoviridae*

Filoviruses are filamentous in shape, pleomorphic in length (up to 14,000 nm), enveloped, and have non-segmented single-stranded negative-sense RNA genomes of about 19 kb in length [1]. The genome encodes seven gene products: nucleoprotein (NP), glycoprotein (GP), RNA dependent RNA polymerase (L), and four proteins termed VP24, VP30, VP35 and VP40 [2,3].

The *Filoviridae* family consists of the genera *Marburgvirus*, *Cuevavirus* and *Ebolavirus*. The genus *Ebolavirus* has five members, namely Bundibugyo virus (BDBV), Ebola virus (EBOV), Reston virus (RESTV), Sudan virus (SUDV), and Taï Forest virus (TAFV) [3].

Filoviruses are categorized among the deadliest zoonotic viruses known to affect human beings with mortality rates reaching up to 90% depending on the viral species and host [4,5]. EBOV, SUDV, BDBV, TAFV and two of the marburgviruses, *i.e.*, Marburg virus (MARV) and Ravn virus (RAVV) sporadically infect humans. The other known ebolavirus, RESTV, appears to be non-pathogenic to humans and the cuevavirus Lloviu virus (LLOV) has only been found in a few individuals of a single cave-dwelling bat species [6]. The appearance of filoviruses and other emerging diseases is often attributed to urbanization with the concomitant invasion of animal habitats, climate change and deforestation, changing socio-economic conditions, increased global connectivity, and changes in biological characteristics of the viral species [7]. The emergence and re-emergence in Africa, the potential for introductions into countries previously free from the disease through human mobility and the international transport of infected animals or animal products make filoviruses a worldwide public health concern [8]. The highest risk for emergence is being seen in tropical countries with high biodiversity [9] and poor health care infrastructure [10], as was evidenced again by the 2013–2015 EVD outbreak of EBOV in western Africa.

Since it was first identified in 1976 in Zaire (now Democratic Republic of Congo; DRC) and Sudan, there have been 26 confirmed EVD/filovirus disease outbreaks in Africa, mainly in the central part of Africa within 10° of the Equator, and more than 67% of the infected individuals have died [11,12]. However, the recent western African EVD outbreak is the largest (both by number of cases and geographical extension) and deadliest recorded so far in medical history [13]. The number of suspected cases and deaths from this single EVD outbreak is already over several times the total of all cases and deaths from known outbreaks in the past 40 years. According to the Center for Disease Control and Prevention (CDC) and World Health Organization (WHO) case reports, it is accountable for 28,646 reported cases and 11,323 deaths as of 27 March 2016 [13].

The source of filoviruses for human index case(s) in most filovirus disease outbreaks is presumptively associated with handling of bush meat or contact with fruit bats. Most human-to-human infections in EVD/filovirus disease outbreaks seem to occur by direct contact with infected patients or cadavers, their blood and body secretions (such as breast milk, sweat) and excretions (such as urine, stool, vomit, semen) [14,15,16,17,18].

Despite almost 40 years of research, filovirus transmission remains incompletely understood and its reservoir elusive. The identification of animal species that can replicate and transmit these viruses to other animals and/or to humans is critical to the development of preventative measures to avert EVD/filovirus disease outbreaks in humans. Moreover, defining the modes of transmission would enable health care workers and communities at large to take the necessary measures in limiting the spread of the disease.

## 2. Pathogenesis

Filoviruses have a broad cell tropism, infecting a wide range of cell types. This tropism is directed by its envelope glycoprotein (GP). Dendritic cells, monocytes, macrophages, endothelial cells, fibroblasts, hepatocytes, adrenal cortical cells, and several types of epithelial cells support the replication of these viruses [19,20,21,22]. Dendritic cells, monocytes and macrophages are preferred as early replication sites of EBOV. Together with neutrophils these cells play pivotal roles in dissemination or trafficking of the virus as it spreads from the initial infection site to regional lymph nodes, probably through the lymphatic system, and to the liver and spleen through the blood. EBOV also affects tissues notably liver, spleen, kidneys, lymph nodes, testes, gastrointestinal mucosa, lungs and skin and causes extensive tissue necrosis [22,23,24]. Infection with EBOV first causes a significant inflammatory response and lymphoid cell apoptosis (most likely due to release of Tumor Necrosis Factor-α (TNF-α)), which leads to lymphopenia [2,25] and suppression of effective adaptive immune response [26]. Moreover, inhibition of the type I interferon response, one of the major anti-viral host defenses, is another important aspect in the pathogenesis of the disease [19,27]. In addition, viral replication in monocytes elicits a storm of pro-inflammatory cytokines. By doing so, the virus disables the immune system, allowing uncontrolled viral replication and dissemination and indirectly damages the vascular system that leads to hemorrhage, hypotension, thrombus and disseminated intravascular coagulation (DIC) followed by shock, organ failure and death [19,27]. Coagulation abnormalities associated with EBOV are not the direct result of virus-induced cytolysis of endothelial cells, and are likely triggered by immune-mediated mechanisms [28]. In experimentally infected animal models or in naturally infected Ebola patients, increased blood concentrations of nitric oxide were associated with mortality [29] because its abnormal production can induce several pathological disorders including apoptosis of bystander lymphocytes, tissue damage and loss of vascular integrity, which might contribute to virus-induced shock [19].

EBOV also causes extensive hepatocellular necrosis with a concomitant reduction in the formation of coagulation proteins. It also affects the adrenal gland and destroys the ability of the patient to synthesize steroids and aggravate the circulatory failure by disabling blood pressure homeostasis [1,2]. In the heart, lungs, intestine and pancreas, impairment of the microcirculatory bed is seen but actual necrotic lesions are rare [30]. In general, the ability of EBOV to disable such major mechanisms in the body facilitates the ability of the virus to replicate in an uncontrolled fashion leading to the rapidity by which the virus can cause lethality [2].

## 3. Transmission

Because of the limited number of filovirus disease outbreaks and associated epidemiological studies carefully examining transmission patterns, conclusions about transmission are based on relatively limited data sets. Moreover most of the inferences are based on retrospective reconstructions of chains of transmission and exposure, and hence there may be inherent recall bias [23].

### 3.1. Source of Infection and Transmission to Index Case(s)

The source of infection for human index cases is usually elusive and not yet properly defined. Filoviruses are believed to be transmitted from wildlife to people through contact with reservoir fruit bats, and through intermediate or amplifying hosts, such as monkeys, great apes, duikers or pigs that have themselves become infected through contact with bat saliva or feces [23,31].

Close contact with the organs, secretions, blood and other bodily fluids of these wild animals can potentially transmit the disease to humans [32,33]. Consumption of freshly killed bats [34], hunting and butchering of chimpanzees, gorillas, duikers and monkeys for food [18,35] are widely accepted as a likely source of infection in rural villages, but concerns exist about the validity of serological results [36]. In many instances, human infections have been preceded by disease in wildlife, and these infected animals acted as either dead-end hosts and/or amplifying hosts [37]. There are also instances where the EVD/filovirus disease outbreak is preceded by huge migration and settlement of bats in the outbreak area followed by massive hunting [34]. Moreover, human displacements of fruit bats through deforestation and roosting behavior of bats may force bats to live closer to—and hence facilitate their contact with—humans [33].

The fact that EVD/filovirus disease outbreaks seldom occur suggests the presence of a rare or ecologically isolated animal reservoir having few contacts with humans and NHPs [38]. Although EBOV was reported for the first time nearly four decades ago, the reservoir of this zoonotic pathogen remains unclear. Despite the finding of EBOV nucleic acid, antigen or antibodies, it has never been isolated from bats or any putative animal reservoir so far [39,40]. Nevertheless, accumulating evidence is identifying fruit bats as the natural reservoir of EBOV. In 2005, the first direct evidence (*i.e.*, viral nucleic acids) was published indicating bats as reservoir hosts for EBOV [40], and research has since been growing to understand the role that bats play in the maintenance, transmission, and evolution of filoviruses [40,41,42,43,44,45,46,47]. The isolation of Marburg virus from wild-caught, apparently healthy, fruit bats [44] further substantiates the assumption that bats are strong reservoir candidates for Ebolaviruses. Antibodies to EBOV have been found in several fruit bat species in Ghana: African straw collared fruit bat (*Eidolon helvum*), Franquet’s epauletted fruit bat (*Epomops franqueti*), Gambian epauletted fruit bat (*Epomophorus gambianus*), Hammer-headed fruit bat (*Hypsignathus monstrosus*) and Veldkamp’s bat (*Nanonycteris veldkampii*). However, serology and virus isolation carried out on 539 bats captured during the 1995 Kikwit EVD outbreak were all negative for antibodies and virus isolation was unsuccessful [40,41,42,43,44,45,46,47].

The means of local enzootic maintenance and transmission of the virus within bat populations remain unknown [41]. Experimental infections of fruit and insectivorous bats showed that filoviruses replicate to high titers in several organs [48]. EBOV nucleotide sequences could still be detected in fecal samples up to three weeks after infection. Furthermore, infected bats did not develop disease signs, which is an important condition to function as a natural host in which the virus might persist. The virus that persists in the reservoir bat species, with little or no transmission, might be sporadically activated through an appropriate stimulus like stress, co-infection, change in food sources, pregnancy and parturition [49] or any, as yet unrecognized, trigger. This hypothesis would explain the sporadic nature and periodicity of filovirus disease outbreaks in Africa [19].

The sources of some other EVD outbreaks like the 1976 Yambuku outbreak, the 1996 and 2004 outbreaks in Gabon and Republic of Congo, and the 1976, 1979, and 2004 outbreaks in Sudan are still unknown [50,51]. This in turn raised the suspicion of additional reservoir and amplifying hosts.

Some publications emphasized the presence of other reservoir hosts in sub-Saharan Africa responsible for the reemerging EVD outbreaks. In this regard, Leroy and colleagues [34] identified *Miniopterus inflatus* (a species among insectivorous bats) as a potential EBOV reservoir. In 1998, EBOV RNA (virus glycoprotein or polymerase gene sequences) was found in six mice (*Mus setulosus* and *Praomys* sp.) and a shrew (*Sylvisorex ollula*) by reverse transcription PCR [51,52]. Although virus isolation was unsuccessful and the results have not been confirmed by other groups, the authors proposed these species as possible reservoir hosts.

Non-human primates, especially gorillas and chimpanzees, can be infected by EBOV and develop highly lethal infection and massive population declines. According to Leroy *et al.* [53] and Harcourt [54], great-ape populations in Gabon and the DRC were devastated by EVD outbreaks, leaving these two species critically endangered in this part of the world. Some ecological data suggest that EBOV has contributed to >80% decline in local great ape populations in Gabon and DRC [35,39,53]. The disease, together with the slow reproductive cycle of the great apes and the customary hunting, poaching and deforestation, also induced fear of extinguishing one of the world’s largest populations of gorillas and chimpanzees in the affected countries [11,37,53]. In this perspective, the linkage between Ebola and great apes, especially gorillas, marks the first time a mammal has been placed on critically endangered list because of an infectious disease [55].

### 3.2. Human to Human (Secondary) Transmission

Secondary transmissions are known to be caused by close contact with infected patients; direct contact (through broken skin or mucus membranes) with infected blood, tissue, or body fluids; or reuse and improper needle and syringe hygiene [56]. Unsafe burials that involve direct contact with dead bodies are widely incriminated as major transmission ways. Practices of traditional healers (including close body contact) who had been treating Ebola patients are also incriminated in some outbreaks, including the western African EVD outbreak [57]. According to Prescott *et al.* [58] viable EBOV can persist for over 7 days on surfaces of bodies, confirming that transmission from deceased persons is possible for an extended period after death. EBOV has been cultured from saliva, breast milk, urine, and semen of infected patients; in addition, viral RNA has been detected by RT-PCR from stool, tears, and sweat and in rectal, conjunctival, vaginal, and skin swabs [59,60,61,62,63]. Infected persons can shed EBOV and Marburg virus for several weeks to months after infection. The virus also can persist in convalescent patients, suggesting that transmission may occur after that symptoms have disappeared [62]. EBOV RNA has been detected from semen up to 101 days after illness onset [16,63], and as for Marburg virus, sexual transmission of EBOV has recently been confirmed [16,17,63,64]. Infectious virus particles have also been isolated from skin, body fluids, and nasal secretions of experimentally infected non-human primates.

Arthropod-borne transmission is theoretically possible, but current evidence suggests it is very unlikely. The attempt made to detect the virus from over 800 bedbugs in DRC at the time of initial investigations [65] as well as the collection of almost 35,000 arthropods during the 1995 Kikwit EVD outbreak [66] revealed a negative result. The experimental trials made by Turell *et al.* [67] and Swanepoel *et al.* [48] to infect arthropods by intrathoracic inoculation of RESTV and EBOV respectively, were also not successful.

While contact with bodily fluids from Ebola patients remains the most likely route of transmission, 18 (6.6%) of the 274 cases in the 1976 SUDV EVD outbreak in Nzara, Sudan and 55 (17.4%) of the 316 cases during the 1995 EBOV EVD outbreak in Kikwit, DRC had no direct physical contact with an infected person or known infected carcass [38,68]. These observations point to other routes of transmission (e.g., human to human respiratory tract infection through droplets and aerosols) or may suggest that other, unidentified animal sources may be involved in EBOV transmission to humans. RESTV EVD outbreaks with documented aersol transmission among non-human primates and asymptomatic infection in humans, as well as experimental infections carried out with EBOV have further raised concerns that—at least some—filoviruses could be naturally transmitted by aerosols [23,69]. However, the aerosol mode of transmission remains highly controversial.

Theoretically, a number of factors affect the likelihood of aerosol transmission of a given pathogen, such as the length of time that particles reside in the air, the velocity and mechanism by which respiratory droplets are propelled from the source, the length of time particles remain infectious once expelled, variations in particle size and density, and issues such as ambient temperature and humidity [23,70]. The other point that should be kept in mind is the difference between airborne and aerosol transmission. The two terms are defined by the Healthcare Infection Control Practices Advisory Committee (HICPAC; www.cdc.gov/hicpac) (see Box 1).

Box 1. **Healthcare Infection Control Practices Advisory Committee (HICPAC) definitions of aerosol and airborne transmission.**

**Droplet (aerosol) transmission** is a form of direct contact transmission in which respiratory droplets carrying infectious pathogens transmit infection when they travel directly from the respiratory tract of the infectious individual to susceptible mucosal surfaces of the recipient, generally over short distances, necessitating facial protection. Droplets traditionally have been defined as being >5 micrometers in size.

**Airborne transmission** is a form of transmission resulting from the inhalation of small respirable particles that remain infectious over time and distance and can be dispersed over long distances by air currents. Droplet nuclei are 5 micrometer or smaller, and are considered to be droplets that have partially evaporated. Droplet nuclei are considered responsible for airborne transmission.

Based on the HICPAC definitions, there are very few theoretical, case-based or research-based articles claiming airborne transmission of filoviruses between humans or NHPs. However, there is limited epidemiological evidence [57,68,71] and some experimental studies supporting aerosol transmission of EBOV, SUDV and RESTV.

### 3.3. Evidence for Natural Aerosol Transmission of RESTV

After its first detection in 1989 (Reston, Virginia) in imported cynomolgus macaques, RESTV was subsequently identified in domestic swine in the Philippines during a naturally acquired co-infection with Porcine Reproductive and Respiratory Syndrome Virus (PRRSV, family *Arteriviridae*, genus *Arterivirus*) and Porcine Circovirus type 2 (PCV-2; family *Circoviridae*) [72,73,74,75]. RESTV causes asymptomatic infection to mild respiratory symptoms and severe mortality in cases of co-infection with other viral pathogens like viruses in the families *Arteriviridae* and *Circoviridae* [72].

Sayama and colleagues [74] tested swine sera using IFAT and IgG-ELISA specific for RESTV-NP and GP, and neutralization tests. Out of 215 swine sera collected in 2008 at two RESTV-affected farms in the Philippines, approximately 70% were positive for RESTV antibodies. On the other hand, none of the 98 swine sera collected in 2010 from Tarlac (a non-epizootic region in the Philippines) and none of the 49 swine sera collected from a slaughter house in Japan (used as a negative control) showed positive reactions in any of the diagnostic tests.

Sequence analysis on microarray captured viral cDNA revealed that three RESTV isolates were divergent from the original virus isolated from macaques’ in 1989. This in turn suggests that RESTV has been circulating since, and possibly before, the initial discovery of RESTV in monkeys exported from the Philippines in 1989 [72].

Another study conducted in China by Pan *et al.* [73] also revealed the presence of RESTV in pigs that died after showing typical clinical signs of PRRSV infection. Out of 137 spleen specimens taken from these pigs and examined for the presence of the virus, 2.92% (*n* = 4) were found to be positive for RESTV by RT-PCR.

To rule out the effect of other pathogens affecting pigs, Marsh *et al.* [75] conducted an experimental study on five-week-old pigs using a 2008 Philippines swine isolate of RESTV. The pigs were exposed via oro-nasal or subcutaneous route and demonstrated viral replication in internal organs and shedding of the virus from the nasopharynx asymptomatically. Although the infected pigs were not showing clinical signs, viral shedding can pose a transmission risk to farm, veterinary, and abattoir workers. The highest levels of virus replication were observed in lung and lymphoid tissue, the tissues from which virus was isolated in the original disease investigation [72]. It has been further hypothesized that preexisting subclinical respiratory infections caused by organisms such as *Mycoplasma* may predispose animals to enhanced RESTV replication as increased populations of alveolar macrophages may support higher levels of virus replication. It is also possible that asymptomatic infections in pigs could contribute to disease severity in the context of other infections such as PRRSV [73]. Although conclusion from these data should be made with caution, the detection of RESTV in domestic swine raises important biosecurity concerns about the potential for disease emergence in humans and other livestock principally in animals for food consumption and perhaps in pets [72,75]. Moreover, the apparent occurrence of human infections (as evidenced by seropositive titers) further increased the concern and worry of researchers, farm owners and the public at large. While RESTV has not been seen to result in any human disease, there is a concern that its passage through swine may allow RESTV to diverge and shift its potential for pathogenicity [72,76]. Moreover, the 15 recorded RESTV-seropositive persons were all adult males [77], and hence the consequences of infection in pregnant women, the very young and old, and the immunocompromised or malnourished individuals could be different [76]. The effective natural transmission of RESTV from infected pigs to naive pigs and monkeys strongly suggest that pigs or pig farming should be considered as potential risk factor in human Ebola. As a precautionary measure they should be considered as potential amplifying hosts until proven otherwise [78]. Moreover, Ebola infection in pigs can pose direct economic consequences certainly due to widespread culling of pigs, as was done during RESTV EVD outbreaks in the Philippines. Similarly, over a million pigs were culled following a Nipah virus outbreak in Malaysia and Singapore with consequent financial losses [79]. Furthermore, consumer fears often have significant economic repercussions illustrated by the avoidance of pork meat consumption [76].

### 3.4. Evidence of Experimental Aerosol Transmission of EBOV and SUDV

Several experimental studies have been conducted on different animal models and offered evidence that Ebola viruses are capable of causing aerosol infection in NHPs and other animals when the animals are: (i) exposed to artificially aerosolized virus [69,80,81]; (ii) exposed via oro-nasal routes with varying amount/dose of virus [80,82]; and (iii) separately housed in proximity with virus-inoculated animals of the same or different species [83,84,85]. Under the following paragraphs the main criteria required for aerosol transmission *vis-a-vis* establishment of the virus in the respiratory system, viral shedding through oro-nasal routes, viral survival in the environment and induction of respiratory pathology/disease in secondary host are summarized and discussed based on experimental studies done so far.

#### 3.4.1. Establishment of the Virus in the Respiratory System

Experimental studies conducted on non-human primates (NHP) by inoculating EBOV via the aerosol route were able to induce fatal disease 5 to 12 days post inoculation. The studies further demonstrated respiratory involvement with multiplication of viral particles within alveolar spaces [22,69,80], within type I pneumocytes and macrophages of the lungs [69], in type II pneumocytes, bronchiolar epithelial cells and endothelial cells [80,83], significant primary and initial infection of lymphoid tissues in the upper and lower respiratory tract and the mediastinal lymph nodes [82]. Similarly viral antigen was also observed within alveolar and septal macrophages, pneumocytes, and epithelial cells of the respiratory tree of cynomolgus macaques housed separately in cages with experimentally infected piglets [84] and in guinea pigs exposed via aerosols to a guinea pig-adapted EBOV strain [82]. Immunohistochemical staining shows viral antigens in alveolar macrophages, endothelial cells, fibroblasts and other interstitial cells [21].

Similarly, experimental infection done by using aerosol doses of 50 or 500 pfu (plaque forming units) of SUDV on groups of cynomolgus macaques, rhesus macaques, and African green monkeys also induced typical disease in all of these NHPs on day 6 post-exposure [81]. Aerosols were artificially generated by a three-jet Collison nebulizer and the animals were then exposed to SUDV in a head-only aerosol chamber within a class III biological safety cabinet. The aerosol dose used by Zumbrun and colleagues [81] was lower than the dose used for intranasal inoculation by other researchers [82,83,84,85]. In line with this, Tellier [86] stated that aerosol dose of influenza virus is considerably lower than the infectious dose by intranasal inoculation and that aerosol inoculation results in more severe symptoms. This difference could presumably be explained by the fact that aerosol particles (especially those about 5 µm) are able to deposit and establish deeper in the respiratory tract where the contact surface area is much larger [87].

#### 3.4.2. Shedding of Ebola Virus through Oro-Nasal Routes

To initiate infection, Ebola viruses must be shed from an infectious person, and must enter cells in a susceptible person, where it can initiate infection [70]. Shedding of EBOV from the mouth and nasal cavity of domestic pigs was detected for up to 14 days following intranasal, intraocular and oral route inoculation [84]. The virus was detected in bronchial washings of rhesus monkeys after 2 days of EBOV inoculation by aerosol route [88]. Johnson and colleagues [69] also detected copious extracellular EBOV antigen in secretions on the mucosal surfaces of the nose, oropharynx and airways of monkeys exposed by intranasal route. The presence of the virus in the respiratory secretions is suggesting a high chance of passing the infection for naive cage mates through the aerosol route [89]. At the very least, the potential exists for aerosol transmission, given that virus is detected in bodily secretions, the pulmonary alveolar interstitial cells, and within lung spaces [81]. Further experiments regarding the generation of EBOV in the respiratory tract and during Aerosol Generating Procedures (AGPs), as well as the stability of EBOV in droplets and on surfaces, could help to define all routes of infection and improve infection control policies [90].

#### 3.4.3. Survival of Ebola Virus Particles in Aerosol Droplets

Following shedding of the virus from the respiratory and other body systems in the form of droplets, the particles should be able to reside in the air and remain infectious till inhaled by susceptible individuals. Very few studies have been conducted to assess the survival of Ebola virus in different environmental conditions. Piercy and colleagues [91] created Ebola-containing aerosols and, on the basis of decay rates, estimated that EBOV and RESTV can survive in aerosols for approximately 100 and 160 min, respectively at 50% to 55% relative humidity and 22 ± 3°C. This result clearly showed that Ebola can remain in the air, perhaps in small amount, for 60–90 min within small droplets even in African climatological conditions. The most important factor for biological decay is the change in water content of the aerosol droplets. Viruses containing structural lipids (such as Ebola) are hydrophobic and generally more stable than lipid-free viruses. Viruses with structural lipids survive best in dry air with a relative humidity less than 50%–70% [92,93]. The amount and length of time that infectious particles reside in the air is dependent on several environmental factors and varies with the particle size and density in the aerosol droplets. According to Brosseau & Jones [94], 3 µm particles can take up to an hour to settle in still air. However, with air currents, these and smaller particles can be transported over considerable distances before they are deposited on a surface.

One study conducted by Sagripanti & Lytle [95] to evaluate the sensitivity of three lethal viruses dried on smooth surfaces to ultraviolet radiation (around 254 nm) revealed 9.5%, 4.5% and 3.5% UV-protected irradiated virus particles for Lassa, Vaccinia and Ebola, respectively. Although the protected virus population corresponds to a relatively small fraction (<10%) of the total viral population, this protected fraction of virus dried on fomites and environmental surfaces could be high in absolute numbers, posing a serious risk to human health within hospital or in the environment contaminated after a natural or intentional viral release.

#### 3.4.4. Induction of Respiratory Pathology and Disease in Secondary Hosts

Naive piglets and NHPs kept in cages where there is presumably no direct contact with those oro-nasally exposed piglets to EBOV were infected and developed microscopic respiratory lesions. The lesions were similar to the ones observed in intentionally exposed cohabitants and includes interstitial pneumonia primarily oriented around terminal bronchioles, inclusion bodies in macrophages [80,82,84,85] and numerous multinucleated cells within alveoli [80]. Considering the pathology of the respiratory system, the dual exposure routes are particularly important in determining the expression of disease in the lung, with distinct patterns of lesions depending upon whether the route of entry is aerogenous or hematogenous. Broncho-interstitial pneumonia, characterized by injury to both the bronchiolar and the alveolar epithelium, is commonly caused by aerogenous viral infections [96]. Moreover, such pathological changes in the respiratory system were generally not noted in primates and laboratory animals inoculated by other routes [20,25,69].

Animals challenged intranasally were more infectious for naive cage mates or other animals housed nearby than animals inoculated intraperitoneally or intramuscularly. This hypothesis is substantiated by the articles published by Wong *et al.* [89], Weingartl *et al.* [84], Twenhafel *et al.* [25] and Alimonti *et al.* [97] who did aerosol infection on guinea pigs, piglets, rhesus macaques and parenteral infection in monkeys, respectively. The respiratory system is unique in its vulnerability to injurious agents including viral particles because of its high surface area and the involuntary nature of ventilation [96]. In line with this, Twenhafel *et al.* [82] stated that the quantity of cells initially infected (*i.e.*, macrophages, dendritic cells) is likely much higher with aerosol challenge compared with intramuscular challenge due to the large surface area of the respiratory tract. Other routes of inoculation generally did not lead to lesions in the respiratory tract comparable to those observed in animals infected through aerosols [85,98].

Although there is strong debate on the potential aerogenic transmission of filoviruses, it should be stressed that the transmission by air is not similar to influenza or other airborne infections. The viral particles are limited in the health care units and affected villages or households having direct or indirect contacts with patient(s), if it was really an airborne virus like influenza it would spread rapidly and involve wider geographic area and population. Based on the existing literature, filoviruses have very little to no capacity to be airborne (*i.e.*, inhalation of infectious particles at a distance from the source). The virus does not transmit from an infected person to a susceptible person that is located at a distance [25,70]. First, the virus will not remain viable by the time it gets to the distant point because the aerosol is already desiccated. Secondly, the viral load or aerosol particles in the air gradually decrease with distance from the source to the extent not sufficient to induce infection. However, Chiappelli *et al.* [33] stated that there is a distinct possibility for EBOV to become airborne because of the customary and high mutation rates of negative sense RNA viruses. According to Brown *et al.* [99] although it is unclear that these mutations carry any fitness advantage or not, EBOV in western Africa is not behaving differently than what has previously been reported [100]. There is no change in route of transmission, no suggestion of airborne spread, no significant differences in disease presentation. Besides, none of the 23 viruses that cause serious disease in humans have been known to mutate in a way that changed their mode of infection [101].

If aerosols containing Ebolavirus were to enter the lungs of uninfected individuals, it is possible that primary pulmonary infections could occur (as shown in animal studies), which could then result in active viral shedding from the respiratory tract, thus potentially setting up a cycle of ongoing respiratory transmission in humans [23,102]. From the experimental work conducted so far, Ebola infection has proven to induce respiratory complications, to shed via the respiratory secretion and to induce similar pulmonary lesions both in animals exposed to aerosols and in those kept nearby in separate cages with no close contact.

An interesting and important question is whether aerosol transmission is involved in animal-human or human-human filovirus transmission?

The three epidemiological studies conducted by Baron *et al.* [51], Roels *et al.* [68] and Francesconi *et al.* [71] partially raised the question of aerosol EBOV transmission because of the occurrence of infection in individuals who had been in the same room but did not have direct contact with the primary case. As discussed earlier, the possibility of aerosol transmission also arose following the 1989 RESTV EVD outbreak at a primate quarantine facility in Reston, Virginia, where aerosol transmission was thought to occur between monkeys and to several animal care workers who didn’t had direct contact. These workers showed antibody levels indicating they had been exposed [74,103].

The limited autopsies performed on humans primarily during the 1976 SUDV and 1995 EBOV EVD outbreaks revealed congestion, focal intra-alveolar edema, diffuse alveolar damage, and hemorrhage in the lungs. Viral inclusions within alveolar macrophages and free viral particles within alveolar spaces were also found [21,22,23] suggesting that infectious aerosols could be emitted from the respiratory tract.

Aerosols emitted from the respiratory tract during coughing, sneezing or talking contain a wide distribution of particle sizes, including many that are small enough to be inhaled [95,104,105]. The droplets are emitted or ‘propelled’ associated with proteins and other materials of the body fluid. Then these droplets will evaporate to smaller particles, losing about 50% of their diameter within a second of release [104]. Microorganism-laden particles suspended in air may be inhaled, deposited in the respiratory tract, and reach cells in which infection can begin. Among the several cell types in which Ebola viruses initiate infection, macrophages and dendritic cells are found abundantly throughout the respiratory system and hence a wide range of particle sizes carrying Ebola virus may deposit anywhere in the respiratory tract and initiate an infection [70].

Cough can be a common symptom in Ebola patients especially during the late phases of the disease when viral loads in serum significantly increase and hence the virus is copiously emitted in most body fluids and as aerosol particles of various sizes [23]. Based on the review made by Osterholm *et al.* [23] prevalence of reported cough is variable in case series, ranging from “rare” to 49%. Moreover, a World Health Organization study on the first 9 months of the epidemic in western Africa found that nearly 30% (194 out of 665) of the patients experienced coughing, and 2.4% (20 of 831) had a bloody cough. A cough in humans is believed to yield approximately 9 × 10^5^ particles, out of which >95%–97% are smaller than 2 µm [105,106]. However, there is still information gap and uncertainty in the amount of virus actually shed by infected humans through these cough particles or in their respiratory secretions.

Apart from coughing, sneezing and talking, virus containing droplets can be emitted from the patient through vomiting, diarrhea and through some medical procedures like suctioning, endotracheal intubation, cough-induction by chest physical therapy, and cardiopulmonary resuscitation [71]. Therefore, inhalation of infectious aerosols near an infected person concomitantly leads to deposition of the pathogen throughout the respiratory system [70]. Piercy *et al.* [91] also underlined that if filoviruses were deliberately or accidentally aerosolized during normal laboratory or clinical practices, they may pose a significant threat to humans, as they are able to remain infectious over a significant period of time.

The risk of transmission of pathogens via droplets is limited to workers within 3 feet of an infectious patient [107]. According to HICPAC, the distance droplets travel depends on the velocity and mechanism by which respiratory droplets are propelled from the source, the density of respiratory secretions, environmental factors such as temperature and humidity, and the ability of the pathogen to maintain infectivity over that distance. Johnson *et al.* [69] emphasized that, although coughing was common among the human Ebola cases in Africa, there was no direct evidence for aerogenic spread of the virus in human populations. The authors mentioned the non-sufficient amount of viral shedding via respiratory route and the hostile ambient temperature in African villages as the main deterring factors to see the contribution of coughing and aerosol transmission in natural Ebola epidemics. However, aerosol transmission is thought to be possible and may occur in conditions of lower temperature and humidity which may not have been factors in EVD/filovirus disease outbreaks in warmer climates [69].

Being pantropic, filoviruses can be ‘opportunistic’ respiratory pathogens as per the classification of Roy & Milton [108] because infectious aerosols (either large or small droplets) may be generated and emitted during the course of the disease. Epidemiological and experimental evidences suggest that viral loads in blood and secretions rapidly increase during the course of illness, with the highest levels of virus shedding observed late in the course of illness [1,2]. Perhaps the most uncertainty about filovirus infection concerns the time, duration, and amount of virus that is shed from infected patients, most particularly with respect to aerosol and droplet transmission and the amount of virus shedding that may occur during early infection. To take appropriate preventive measures, this important question needs to be better clarified, especially using sensitive laboratory techniques and referencing these with the very early signs of disease in infected patients.

EBOV RNA was identified with the help of RT-PCR from the outer surfaces of 3 out of 16 apparently clean masks worn by health care workers (unpublished data cited in [23]). Although it does not necessarily indicate the presence of viable infectious viral particles, this finding can strongly suggest either the presence of aerosols in the patient-care environment or cross-contamination of the masks [23]. This finding should warn the community and particularly health care workers, when doffing contaminated masks or other Personal Protective Equipment (PPE) because the infectious dose for ebolaviruses to cause infection in humans appears to be extremely low with 10 or fewer viral particles sufficient to cause infection [109,110].

In general, apart from the above speculations and theoretical arguments, there is no strong evidence of secondary transmission by the aerosol route in African filovirus disease outbreaks purely based on epidemiological data. Moreover, on the basis of epidemiological evidence it is very difficult to definitively demonstrate or rule out aerosol infection, since those individuals exposed to infectious aerosols are also most likely to be in close proximity to and in direct contact with an infected case.

## 4. Conclusions and Recommendations

The transmission route from reservoir to domestic animals and humans is unknown. During human EVD/filovirus disease outbreaks, human-to-human transmission likely occurs mainly through direct skin contact (including sexual contact), contact with contaminated secretions and excretions or accidents with contaminated needles.

Experimental studies conducted with pigs suggest that the respiratory mucosa is a sole participant in multiplication and subsequent transmission of virus, especially in pigs exposed via the aerosol route. There were relatively high viral loads in the upper airway and replicating in macrophages, pneumocytes, and endothelial cells in the lung parenchyma to high concentrations. Moreover, the efficient transmission of the disease to naive pigs cohabiting with infected mates and to NHP may strongly support the fact that pigs are capable of shedding relatively high viral loads into the environment. Therefore, veterinarians, animal handlers and farmers should be advised to take precautions to avoid animal bites, use sharps with caution and to wear proper personal protective equipment (at least gloves, gowns, mouth and eye protection), especially when pigs are involved. In addition, proper decontamination, removal and disposal of personal protective equipment is essential to avoid accidental infection and imply awareness campaigns and training. Veterinarians, animal handlers and pig farmers should also be a primary target population for vaccination, as soon as a protective vaccine is found efficacious. Vaccinating wild animals and vast numbers of domestic animals is challenging and it is unclear if, and how long, immunity would last in these populations.

With the growing experimental evidence and arguments, there is no reason to underestimate the possibility of aerosol mode of transmission, and pigs as a potential amplifying host. The amplification of the virus in piglets and transmission to naive animals housed nearby suggests an urgent action is needed because pigs in most African countries are free ranging/scavenging and are often an essential source of protein. Therefore, there must be a comprehensive epidemiological surveillance of the disease in domestic pigs owned or managed by EVD-affected families, during and after EVD outbreaks, together with contact tracing and rumor-verification of suspected cases or deaths in the community. Moreover, public health and veterinary authorities should create awareness to the communities regarding the housing of pigs and the possible measures they should take when pigs show signs of respiratory complications. Effort also should be made to reduce infection in domestic pigs from a possible wildlife reservoir.
